# Innovative functional polymerization of pyrrole-N-propionic acid onto WS_2_ nanotubes using cerium-doped maghemite nanoparticles for photothermal therapy

**DOI:** 10.1038/s41598-021-97052-6

**Published:** 2021-09-23

**Authors:** Tzuriel Levin, Yakir Lampel, Gaya Savyon, Esthy Levy, Yifat Harel, Yuval Elias, Moshe Sinvani, Iftach Nachman, Jean-Paul Lellouche

**Affiliations:** 1 Institute of Nanotechnology and Advanced Materials and Department of Chemistry, Faculty of Exact Sciences, Bar-Ilan University, 5290002 Ramat Gan, Israel; 2 School of Neurobiology, Biochemistry and Biophysics, George S. Wise Faculty of Life Sciences, Tel Aviv University, 6997801 Tel Aviv, Israel; 3 Faculty of Engineering and the Institute for Nanotechnology and Advanced Materials, Bar-Ilan University, 5290002 Ramat Gan, Israel

**Keywords:** Cancer, Chemistry, Materials science

## Abstract

Tungsten disulfide nanotubes (WS_2_-NTs) were found to be very active for photothermal therapy. However, their lack of stability in aqueous solutions inhibits their use in many applications, especially in biomedicine. Few attempts were made to chemically functionalize the surface of the NTs to improve their dispersability. Here, we present a new polymerization method using cerium-doped maghemite nanoparticles (CM-NPs) as magnetic nanosized linkers between the WS_2_-NT surface and pyrrole-N-propionic acid monomers, which allow in situ polymerization onto the composite surface. This unique composite is magnetic, and contains two active entities for photothermal therapy—WS_2_ and the polypyrrole. The photothermal activity of the composite was tested at a wavelength of 808 nm, and significant thermal activity was observed. Moreover, the polycarboxylated polymeric coating of the NTs enables effective linkage of additional molecules or drugs via covalent bonding. In addition, a new method was established for large-scale synthesis of CM-NPs and WS_2_-NT-CM composites.

## Introduction

About three decades ago, Tenne et al. published a research on polyhedral and cylindrical tungsten disulfide^1^ after many years in which polyhedral and cylindrical structures were found mostly in carbon materials such as carbon nanotubes (CNTs). It was commonly believed that a closed structure exists only in carbon because of the high strain in other materials. WS_2_ inorganic nanotubes (WS_2_-NTs) consist of layers, which are attached to each other by Van der Waals interactions. Each layer resembles a sandwich where a tungsten atom lies between six sulfur atoms, three above and three below, resulting in a hexagonal structure^2^. This three-layer structure is typical to a family of compounds called transition-metal dichalcogenides (TMDCs), which also includes MoS_2_ and WSe_2_ heterostructures^3^. Other interesting inorganic nanomaterials include layered black phosphorus nanosheets with promising optoelectronic properties^3^, nanosheets of its analogue tin monosulfide (SnS) with many potential applications including photovoltaics and optical modulation^4^, nanosheets, NTs and other nanostructures of bismuth^5^ and selenium^6^ with myriad applications including sensors, energy storage and biomedical applications, and Te@Se roll-to-roll NTs for photoelectrochemical-type broadband photodection^7^.

Usually, the diameter of WS_2_-NTs is on the nanoscale, around 100 nm, although Chen et al. reported that they obtained nanotubes with diameter < 10 nm^8^. The length extends to 15 µm. WS_2_-NTs have good mechanical properties such as a high Young’s modulus, in the 150–170 GPa range, and good tensile strength between ~ 4 and 16 GPa, with a maximal elongation of 5–14%^9^. They can withstand 21 GPa shock waves^10^. The TMDC family, and WS_2_-NTs in particular, have low toxicity and good biocompatibility, in contrast to CNTs^11^.

Due to their lack of dispersion in most polar and nonpolar solvents, an effective functionalization is required before using WS_2_-NTs in any relevant application. Only few publications describe WS_2_ functionalization. Raichman et al. used a Vilsmeier-Haack reagent that covalently bonds to the sulfur atoms to obtain a polycarboxylated coating^12^. Based on this work, WS_2_-NTs were coated by a polythiophene shell using 2,2′-bisthiophene-4-carboxaldehyde^13^ or 3-thiopheneethanol linkers, which were covalently attached to the carboxylic groups^14^. Alternatively, a humin-like coating may be applied, which is based on Lewis acid-activated thioglycosylation chemistry that presents conformal and controlled thickness^15^. Tahir et al. also showed that a polymer coating with nitrilotriacetic acid side chains enables chemical attachment onto nanotubes and also serves as a chemical anchor for the binding of histidine-tagged proteins^16^. In addition to polymers, WS_2_-NTs can also be coated with nanoparticles (NPs) to enhance their properties. For example, WS_2_-NTs were decorated with cobalt NPs for photocatalysis^17^. In previous work, WS_2_-NTs were innovatively decorated with superparamagnetic, highly dispersed hydrophilic cerium-doped maghemite NPs (CM-NPs), resulting in strongly magnetic NTs with higher stability in aqueous solution compared to uncoated nanotubes^18^.

The CM-NPs were intensively examined for numerous applications such as magnetic storage, biosensing applications, magnetic separation, drug delivery, cancer hyperthermia, magnetic resonance imaging^19^, and gene silencing^20^. In addition to their biocompatibility and biodegradability benefits^21,22^, cerium-doped maghemite NPs can be linked to various biomolecules such as enzymes, antibodies, or nucleotides, as well as to polymers^23^. This is done by coordination bonding to the cerium atoms that are doped on the NP surface. Here, we specifically utilize these CM NPs on the NT-WS_2_ surface to establish an effective polymer coating. Moreover, our previous work also showed that the WS_2_-NT-CM functionalized composite had enhanced photothermal activity compared to uncoated non-functional NTs owing to their greater stability. The photothermal therapy (PTT) is active at 700 nm, but at this wavelength, tissue penetration depth constitutes a limiting factor for the effectiveness and applicability of these composites. The penetration depth in human tissue increases as the wavelength increases, therefore there is a critical need to incorporate into the NTs a substance that may be active at a longer wavelength in order to improve the beam penetration depth. Accordingly, the NTs were coated with an N-substituted polypyrrole polymer.

**Figure 1:**
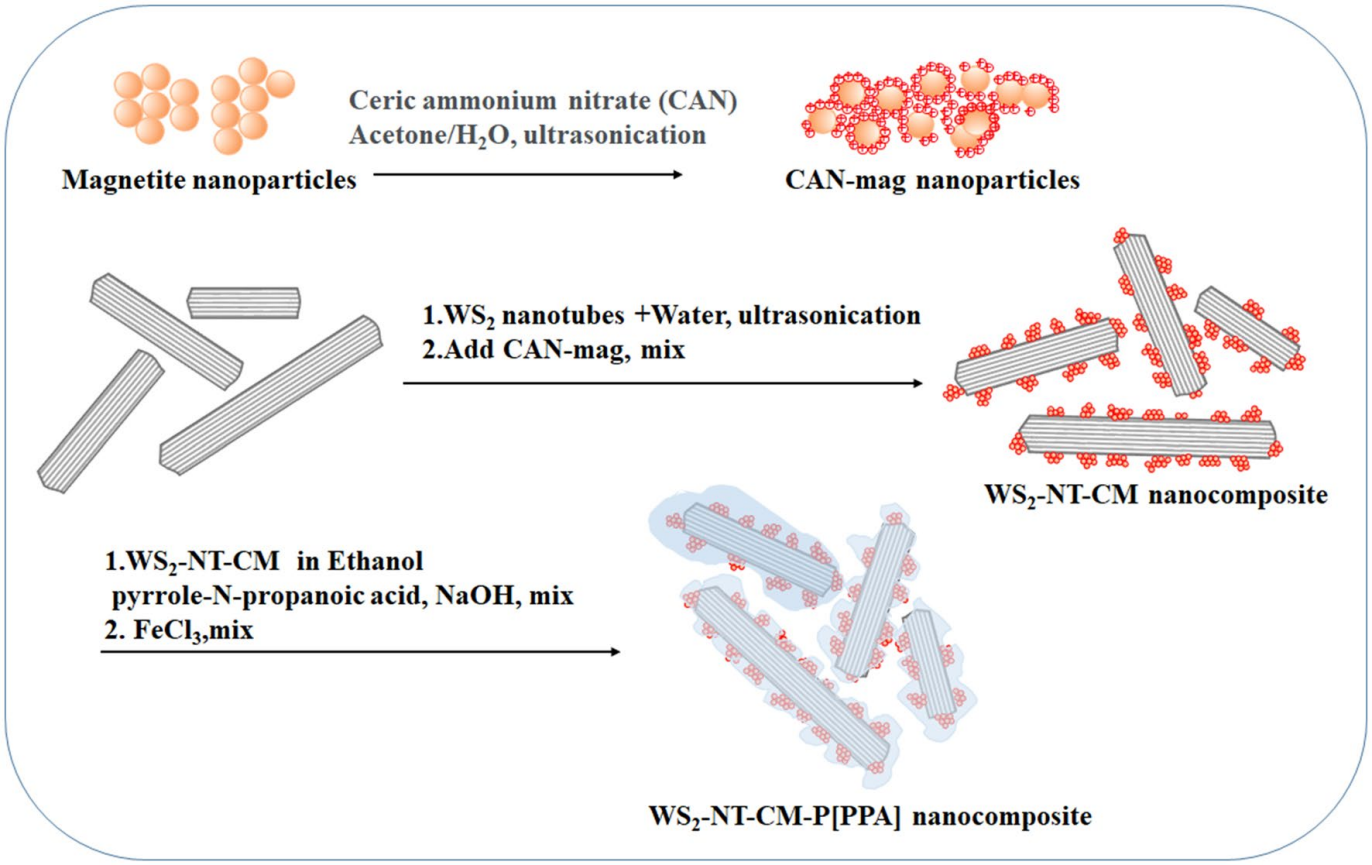
Schematic description of WS_2_-NT-CM-P[PPA] nanocomposite preparation.

As is known, polypyrroles (PPy) exhibit photothermal activity at 808 nm^24–26^, which makes them suitable for increasing the NT-based PTT in deeper tissues. Also, among the numerous conducting polymers, PPy are by far the most extensively studied due to their ease of synthesis, good redox properties, stability in the oxidized form^27^, nontoxicity, and good biocompatibility^28–30^. In medicine, PPy are used for applications in drug delivery systems, biosensors, templates for regeneration of nerve pathways, and tissue engineering^31,32^. In this case, a functional pyrrole–N-propionic acid (PPA) was chosen for two reasons: first, the carboxylic group facilitates the grafting of the pyrrole monomers onto the NTs by coordinately bonding with cerium on the decoration of functionalizing maghemite NPs, thus creating anchor sites for the monomers prior to polymerization. Second, the polycarboxylated coating enables linkage of medicinal and/or biological materials onto the surface of the NTs, thereby further expanding their role from merely photothermal agents to thermoresponsive drug delivery systems. A facile method for polymerization of a functional pyrrole monomer onto WS_2_-NT-CM was thus established by using PPA monomer, as shown in Fig. 1. This innovative WS_2_-NT-CM-P[PPA] composite is a triple-phase structure with an inorganic core, a medial metal oxide decorating phase, and an organic polymer shell with an improved positive zeta potential of + 36 mV, indicating greatly improved water-based stability. To test the effect of the polymer on the PTT activity of this anticipated composite, we examined human HeLa (cervical cancer) cells after incubation with WS_2_-NT-CM and WS_2_-NT-CM-P[PPA] following irradiation at 808 nm.

## Experimental section

### Preparation of CAN-mag (CM) nanoparticles in large quantities

The synthesis is similar to the one presented recently^18^, differences are highlighted in boldface. A solution of FeCl_3_·H_2_O (960 mg, 3.6 mmol) in degassed ddH_2_O water (20 ml) was mixed with an aqueous solution of FeCl_2_·4H_2_O (390 mg, 1.8 mmol, 20 mL H_2_O). The mixture was kept under nitrogen and ultrasonicated for 1 min at room temperature. Then, a concentrated (28–30 wt.%) NH_4_OH solution (2.4 ml) was added, resulting in the immediate formation of a black precipitate of magnetite (Fe_3_O_4_) particles. Sonication was continued for an additional 10 min. The liquid was decanted with the help of magnetic separation, using a 0.5 Tesla magnet. The particles were washed with three portions of ddH_2_O (50 mL each) to neutrality. Then, ddH_2_O (50 ml) was added, and the maghemite NPs suspension was set aside for a minimum of 1.5 h at ambient temperature for aging prior to use.

A solution of CAN (2.00 g, 3.648 mmol) in acetone (24 ml) was added to the decanted magnetite NPs, followed by the addition of degassed purified water (96 ml). The resulting mixture was ultrasonicated while stirring for 35 min at 24% amplitude under nitrogen using a high-power sonicator. The acetone and most of the water were removed by rotary evaporation to a final volume of less than 50 ml. The solution was then centrifuged at 4000 rpm for 5 min to remove the liquid, and the residue was redispersed in ddH_2_O, after which the dispersion was transferred into 50 ml Amicon® Ultra-15 centrifugal filter tubes (100 kD, Millipore, Cork, Ireland). The contents were washed with three portions of ddH_2_O (10 ml each) and centrifuged at 4000 rpm for 10 min at 25 °C each time. The washed nanocomposite was dispersed in ddH_2_O (50 ml). The Fe concentration in the dispersion, determined by atomic absorption (AA), was 5.9 mg/ml.

### Large-scale synthesis of WS_2_-NT-CM nanocomposit

The synthesis is similar to the one presented in the previous article^18^ with differences highlighted in boldface. WS_2_-NTs (200 mg, Nanomaterials Ltd., Yavne, Israel; lot number: TWPO-MA018) were dispersed in ddH_2_O (250 ml) using an ultrasonic probe (set to reach 17.5 kJ, with 20% amplitude) while stirring for 20 min at room temperature. Aqueous CAN-mag dispersion equivalent to 20 mg Fe was added (1:10 Fe/WS_2_-INTs wt. ratio). The mixture was shaken for 48 h at 25 °C, and the resulting WS_2_-NT-CM was separated from the solution using a 0.5 Tesla magnet and washed with three 40 ml portions of ddH_2_O.

### Preparation of WS_2_-NT-CM-P[PPA] nanocomposites

12.80 mg of CAN-mag-decorated tungsten disulfide nanotubes (WS_2_-NT-CM) were stirred in 50 ml degassed ethanol. 40.0 mg of pyrrole-N-propanoic acid (PPA, 287.5 mmol) were dissolved in 5.0 ml degassed ethanol together with 11.50 mg NaOH (287.5 mmol). The PPA solution was added to the NTs and stirred for 2 h under nitrogen atmosphere and away from light, and 11.10 mg of FeCl_3_*6H_2_O (41.07 mmol) were dissolved in 5 ml degassed ethanol and added drop by drop to the reaction mixture. The reaction mixture was stirred for 18 h at room temperature. The composites were separated from the supernatant by magnet-assisted decantation, and washed with ethanol (3 × 30 ml) and ddH_2_O (30 mL). Finally, 27.5 ml of ddH_2_O were added to the composites and placed in an ultrasonic bath for 1 min.

### Characterizations

The composites were characterized similar to the previous article^18^. Briefly, AA was used to determine the concentration of iron (AAnalyst 400 AA Spectrometer, Waltham, MA, USA). Concentrated hydrochloric acid (1000 µL, DaeJung, Busan, Korea) was added to 1 ml composite diluted to 10 mL with ddH_2_O, and set aside overnight for decomposition. The solution was then filtered through a 0.22 µm PTFE syringe filter (Millipore, Darmstadt, Germany).

Transmission electron microscopy (TEM) images were acquired by a JEM-1400 microscope (JEOL Inc., Peabody, MA, US) equipped with a 2 × 2 k CCD camera (Gatan, Pleasanton, CA, US). Samples for TEM analysis were dispersed in water. A drop of the dispersion was placed on a formvar/carbon film on a 400-mesh copper TEM grid (FCF400-Cu, Electron Microscopy Sciences, Hatfield, PA, US) and dried at ambient temperature for 24 h^18^.

Thermogravimetric analysis (TGA) was performed with a TGA/DSC1 analyzer (Mettler-Toledo, Greifensee, Switzerland). All thermograms were recorded in a nitrogen (50 ml/min) environment at a heating rate of 10 °C·min^−1^ over the temperature range of 30–800 °C. Weight change and heat flow were measured simultaneously during the analysis. The results were processed using STARe evaluation software (Mettler-Toledo, Greifensee, Switzerland)^18^.

ATR-FTIR spectra were obtained on a Nicolet iS10 FT-IR spectrometer (Thermo Scientific, Waltham, MA, US) equipped with an iD5 ATR accessory featuring a laminated diamond crystal. Samples were analyzed without further preparation. The data processing was performed using OMNIC 9 spectra software (Thermo Scientific, Waltham, MA, USA).

Ultraviolet–visible (UV–Vis) spectra were obtained on a Cary 100 Bio UV–Vis spectrometer (Agilent Technologies, Santa Clara, CA, USA). Samples were dispersed in water (ca. 0.1 mg/ml).

The temperature profiles for irradiated WS_2_-NT-CM-P[PPA] solutions were measured using a radiometric thermal imaging camera with 320 × 240 pixels, temperature sensitivity of 0.07 °C and spatial resolution of 0.5 mm (FLIR Systems Inc, Boston, MA, model A325). To characterizes the photothermal properties of this nanocomposite, concentrations of 20, 50, 100, 200 and 500 ppm were used. Double distilled water (ddH_2_O) was used as a negative control. For each sample, 1 ml was placed into a well with an rea of 2.0 cm^2^. The laser beam was directed at the sample from above, with a diode laser at wavelength of 808 nm (custom built), with maximum output of 6 W. In each experiment, the laser intensity on the place of the sample was divided by the laser spot area on the sample (0.64 cm^2^). For each experiment, a few seconds of ambient temperature were recorded before irradiating the sample for 120 s.

Zeta potential and dynamic light scattering (DLS) measurements were performed using a Zetasizer Nano-ZS device (Malvern Instruments Ltd., Worcestershire, UK). Samples for zeta potential and DLS measurements were dispersed in water (ca. 0.5 mg/ml).

For photothermal therapy (PTT) experiments, we tested a human cancer cell line (HeLa, ATCC, Manassas, VA, USA). Cells were cultured on 24-well glass plates. When the cells reached 80% confluence, freshly prepared aqueous dispersions of WS_2_-NT-CM-P[PPA] or WS_2_-NT-CM (45 µL, 0.8 mg/ml of WS_2_ component calculated according to an elemental analysis of sulfur) were added to two of the plates, and a third plate, with no additives, was used for control. After 14 h of incubation, the cells were washed three times with PBS buffer, and fresh DMEM medium was added. For each condition, ten representative frames were imaged under a Zeiss LSM7 inverted two-photon microscope at 10 × magnification in phase-contrast. Next, a square region of 230 µm × 230 µm in the middle of each frame was irradiated with an 808 nm laser (Chameleon Vision II) at 90 mW for 31 s. A dye exclusion test of cell viability was performed using trypan blue for staining. A mixture of trypan blue solution and PBS (1:1 v/v) was added to all the wells after laser irradiation. The same frames were imaged after 5 min^18^.

## Results and discussion

The fabrication of WS_2_-NTs coated with polypyrrole (PPy) involves two steps. First, the CM NPs were attached to the NT surface. In the second step, a direct polymerization on the composite surface was implemented due to the step of PPA monomer adsorption and coordinative attachment to the CM NPs (2 h), followed by in situ polymerization by adding the oxidation reagent.

**Figure 2:**
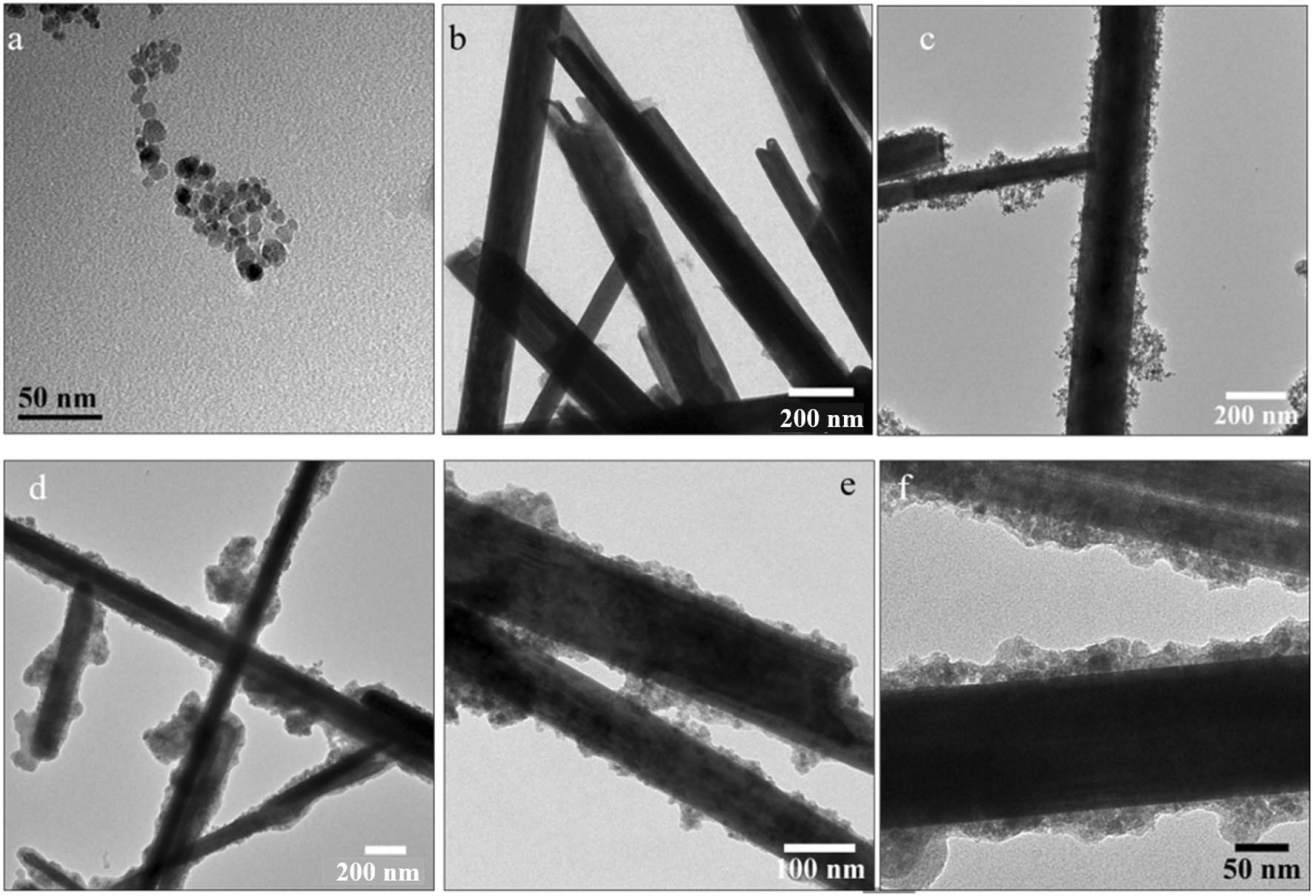
TEM images of (**a**) CM NPs; (**b**) WS_2_-NTs; (**c**) WS_2_-NT-CM; (**d**–**f**) WS_2_-NT-CM-P[PPA].

Transmission electron microscopy (TEM) is a very effective tool for tracking the various stages of surface engineering. At the end of each of the two stages of the composite fabrication, we used TEM to test the morphology. The TEM image of WS_2_-NT-CM (Fig. 2c) show that CM NPs (TEM image of only CM NPs, Fig. 2a) attached onto the WS_2_-NTs in small clusters (S-based coordinative chemical linkage).

Also, a smaller aggregation level of the obtained composites can be observed in comparison to the “naked” untreated starting WS_2_-NTs (Fig. 2b), even though the synthesis was doneon a large scale. The images of the functional composite WS_2_-NT-CM-P[PPA] (Fig. 2d–f) show the polymeric coating around the nanotubes, more precisely around the CM NPs, demonstrating their role as anchors or linkers between the polymer and the WS_2_-NTs.

**Figure 3:**
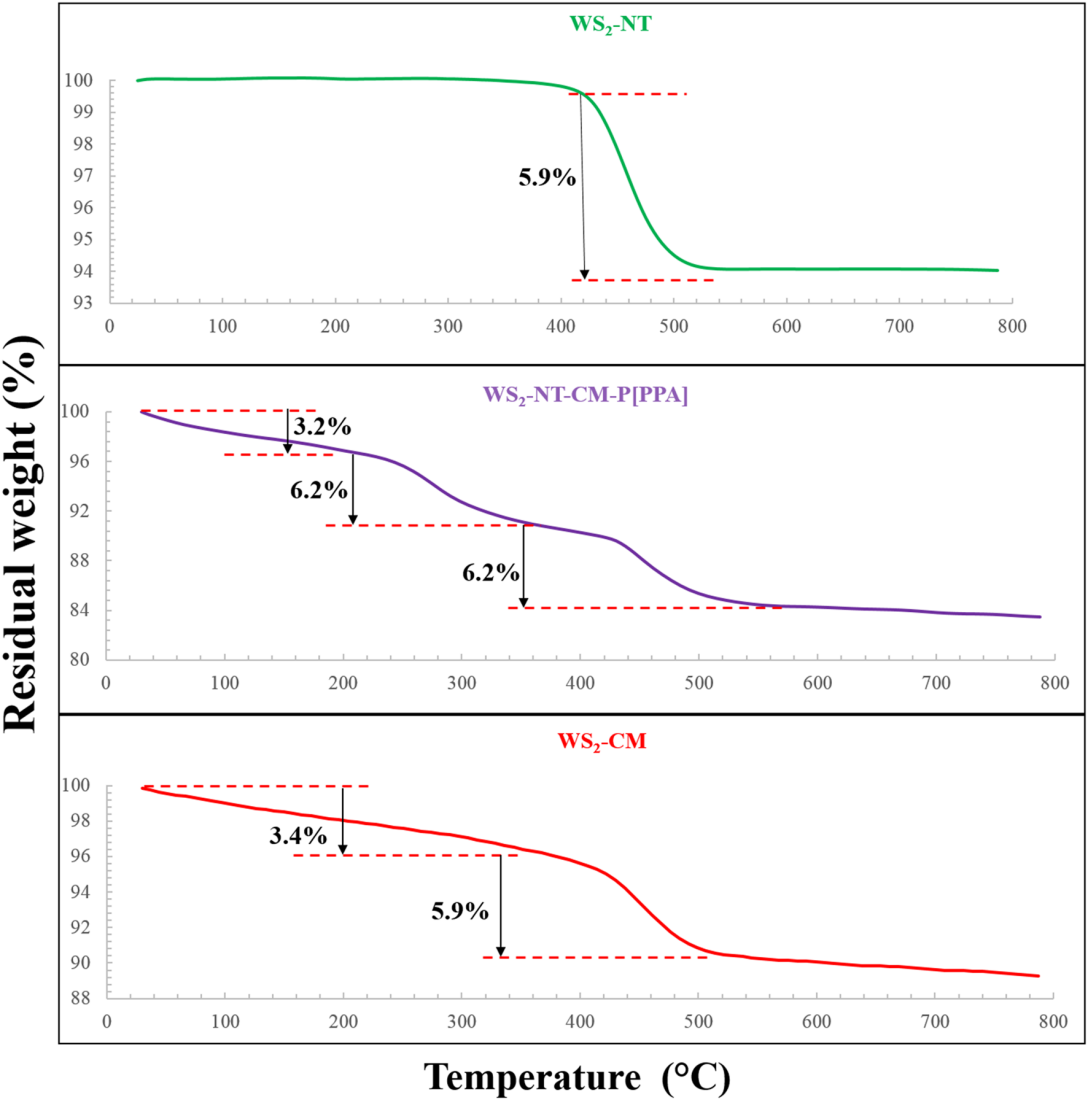
TGA analysis of untreated WS_2_-NTs, CAN-mag-decorated WS_2_-NTs (WS_2_-NT-CM), and CAN-mag-decorated WS_2_-NTs with poly-PPA coating (WS_2_-NT-CM-P[PPA]).

In order to quantify the polymer coating, TGA analyses were performed using a temperature profile of 30–800 °C at 10 °C/min under an airflow of 50 ml/min (Fig. 3). In the temperature range of 120–800 °C, the total weight loss of the untreated WS_2_-NTs, WS_2_-NT-CM and functional WS_2_-NT-CM-P[PPA] was 6.0, 9.5, and 14.6%, respectively.

The untreated WS_2_-NTs exhibited a weight loss of 5.9% in the range of 400–520 °C due to the oxidation of WS_2_ to WO_3_ and the evolution of SO_2_. The nanotubes functionalized with CAN-mag (WS_2_-NT-CM) exhibited a continuous and moderate weight decline in the range of 30–354 °C. This weight loss is related mainly to the release of H_2_O, but also to the decomposition of adsorbed organic materials, and possibly to the scission of the Ce-ligands with their consequent release as nitrogen oxides. A weight loss of 5.9% was observed in the temperature range of 354–520 °C, consistent with the oxidation of WS_2_ to WO_3_ and with the subsequent release of SO_2_.

WS_2_-NT-CM-P[PPA] exhibited the same moderate weight decline in the low temperature range as WS_2_-NT-CM, as well as the characteristic weight loss of 6.2% in the range of 385–580 °C. More importantly, it exhibited a peak weight loss of 6.2% in the temperature range of 206–385 °C, attributed to polymer decomposition and release of combustion products.

**Figure 4:**
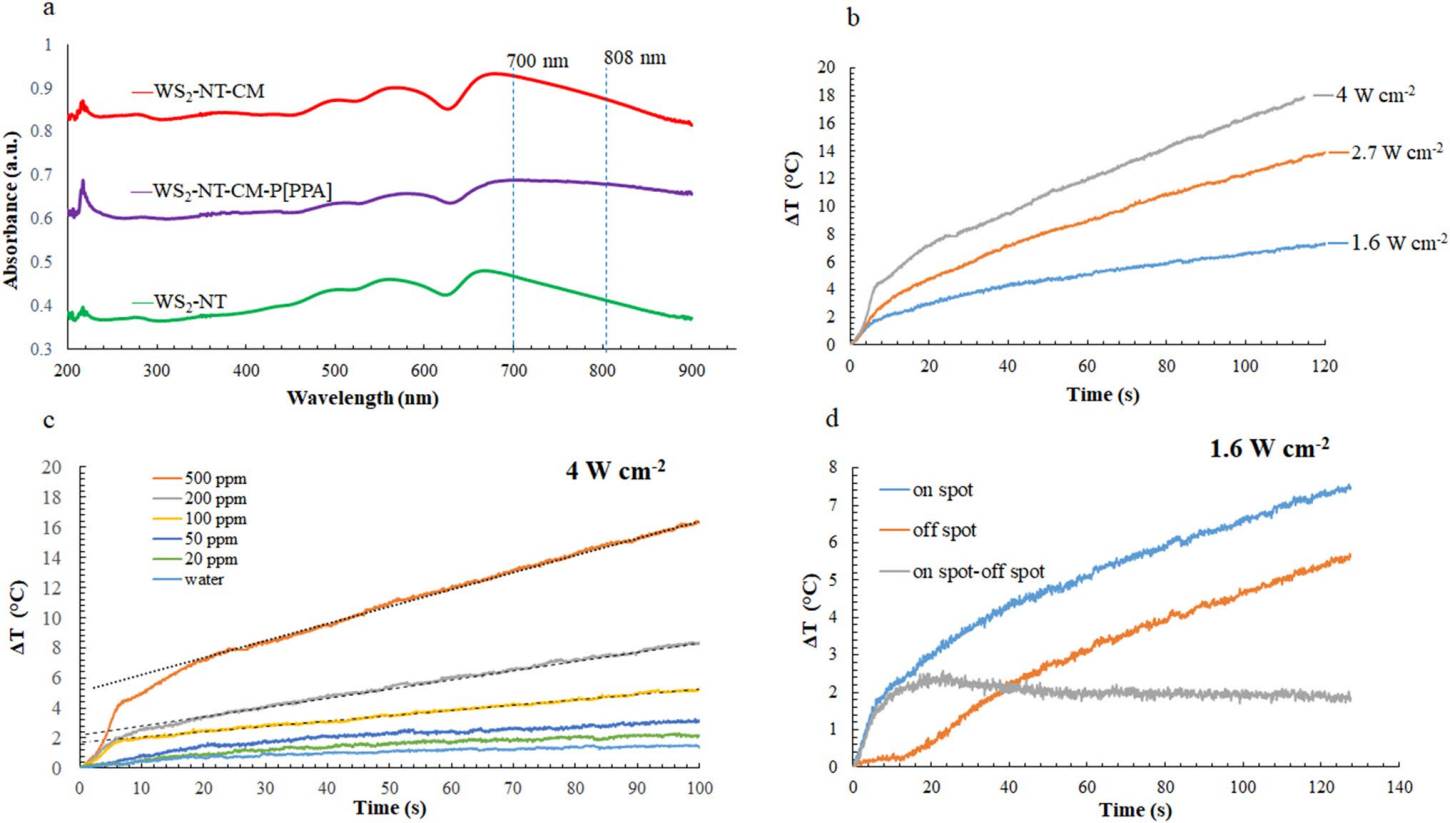
Characterization of photothermal performance of WS_2_-NT-CM-P[PPA]. (**a**) UV–Vis absorbance spectra of untreated WS_2_-NTs and their modified nanocomposites; (**b**) photothermal temperature increase for different radiation intensities (500 ppm); (**c**) photothermal temperature measurement of the irradiated spot on the sample; (**d**) photothermal temperature increase on (blue line) and off (orange line) the spot and the difference (gray line).

As mentioned in the introduction, light absorbance in the near IR range is a fundamental property that indicates the potential to exert a photothermal effect. In this work, UV–Vis spectrometry was performed on the three types of WS_2_-NTs—untreated, CAN-mag (CM)-decorated, and PPA-polymerized—in order to measure and compare their IR absorbance, thus predicting their photothermal activity (Fig. 4a). As the diagram shows, all three composites—WS_2_-NTs, WS_2_-NT-CM, and WS_2_-NT-CM-P[PPA]—demonstrated absorbance peaks around 700 nm originating from the WS_2_-NT core, consistent with our recent results^18^, which demonstrated photothermal activity at 700 nm in WS_2_-NTs and WS_2_-NT-CM. As can be seen for these polymer-free composites, the absorbance declines significantly above 700 nm, rendering them inactive in the deeper penetrating, higher wavelength region of 800–900 nm. However, in the case of the polyPPA coated NTs (WS_2_-NT-CM-P[PPA]), absorbance remained almost the same around 800 nm, and only a minor decline was measured around 900 nm. This observed prolongation of the absorbance range toward 900 nm is attributed to the PPA coating and suggests the possibility of photothermal activity in deeper tissues, while utilizing lower energy (and thus less harmful) light beams.

To characterize the photothermal activity of WS_2_-NT-CM-P[PPA], a radiometric thermal imaging camera was used to trace the temperature elevation at the irradiated spot. Temperature elevation of 500 ppm nanocomposite samples were measured at different laser intensity of 1.6, 2.7 and 4 W/cm^2^ (Fig. 4b), indicating a nice correlation between the heating profile and the laser intensity. Figure 4c shows the temperature profiles for irradiated solutions as a function of time for different concentrations of the WS_2_-NT-CM-P[PPA] composite at constant intensity (4 W/cm^2^), showing again a positive correlation between the NT concentrations and the temperature elevation. For the highest concentration (500 ppm), a temperature difference of about 16 °C was observed. It can be seen that all graphs have two slopes; the first one is sharper and related to the instant temperature rise of the laser spot on the sample. The second slope represents the temperature rise of the entire sample volume due to heat diffusion from the laser spot area, and it reaches steady state (linear temperature rise) 20 s after the laser application. This is confirmed in Fig. 4d where the temperature rise was measured in two points, on the spot area and on the surrounding simultaneously; one can see that both curves rise in parallel after ~ 20 s (see also the difference curve).

Moreover, the photothermal conversion efficiency (PTCE) of the WS_2_-NT-CM-P[PPA] composite was calculated based on the rate of heat absorbed by the water and relative to the laser intensity, leading to efficiency of 33.2%, very similar to the value found in the literature regarding WS_2_ nanosheets (32.8%)^33^. Similar PTCE was obtained by black phosphorus quantum dots (BPQDs)—up to 28.4%^34^. In vitro experiments showed that at a low concentration (50 ppm), BPQDs generated sufficient heat to kill tumor cells almost completely under irradiation with an 808 nm laser^35^, and were successfully combined with immunotherapy^36^. Recently, successful nanomaterials such as tin-sulfide nanosheet-based dual-therapy nano-platforms (SDTNPs)^37^, gold nanoparticles^38–40^ and 2D titanium nanosheets (TiNSs)^41^ showed even higher photothermal performance owing to localized surface plasmon resonances. In the latter case, an exponential temperature increase is evident (see Fig. 3D in Ref^41^), while the gold nanoparticles exhibit both a two-slope (see Fig. 3 in Ref^39^) and a largely linear behavior (see Fig. 3 in Ref^38^).

**Figure 5:**
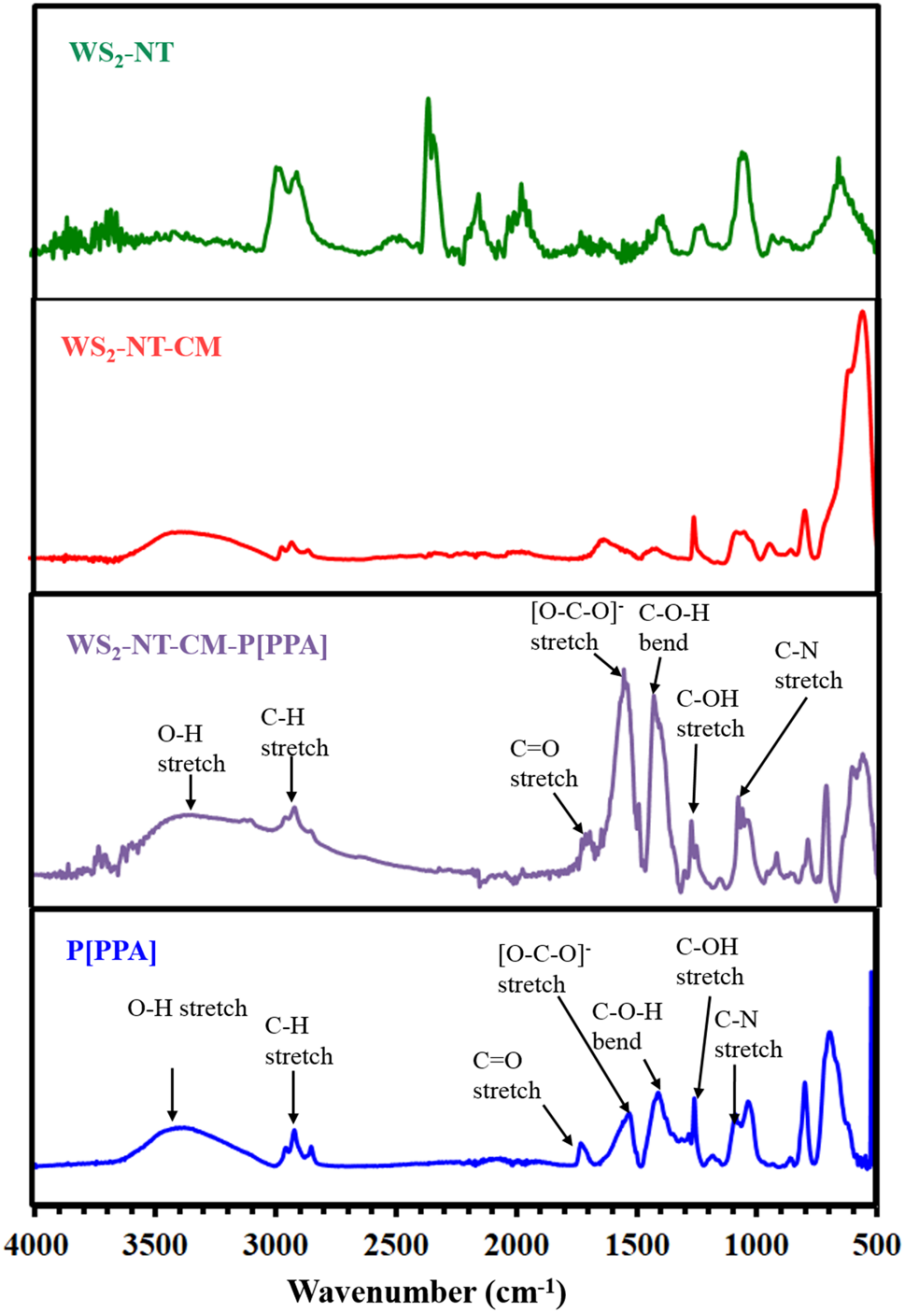
FTIR absorbance spectra of WS_2_-NT, its nanocomposites, and polyPPA (P[PPA]).

Figure 5 shows the FTIR absorbance spectrum of each stage of the synthesis of the targeted composite. In addition, it shows the spectrum of the PPA polymer, which was prepared separately under the same composite polymerization protocol. A full characterization of WS_2_-NT and WS_2_-NT-CM can be found in our previous article, indicating the presence of the CM NPs in the WS_2_-CM composite^18^. Our discussion will focus on the spectrum of WS_2_-NT-CM-P[PPA] and P[PPA]. A broad band around 3400 cm^−1^ corresponding to the peaks of the O–H group stretching vibration is seen in both WS_2_-NT-CM-P[PPA] and P[PPA] composites. The peak at 2930 cm^−1^ is attributed to aliphatic C-H stretching. The bands at 1730 cm^−1^ in P[PPA] and the shifted band at 1700 cm^−1^ in WS_2_-NT-CM-P[PPA] are related to the C=O stretching vibration of the saturated carboxylic acids groups and indicate that the C=O bonding is enhanced for the WS_2_-NT-CM-P[PPA] composites. The peaks at 1580 cm^−1^ in P[PPA] and 1600 cm^−1^ in WS_2_-NT-CM-P[PPA] are characteristic of the COO^−^ (carboxylate) vibrational mode. The peaks at 1437 cm^−1^ in P[PPA] and 1411 cm^−1^ in WS_2_-NT-CM-P[PPA] correspond to their carboxylic acid C–O–H bending. The peaks at 1260 cm^−1^ in P[PPA] and 1282 cm^−1^ in WS_2_-NT-CM-P[PPA] are attributed to carboxylic C–C–OH stretching, and the peak at 1090 cm^−1^ is specifically related to the C-N side chain stretching. The FTIR results strengthen the claim that the triple phase composite WS_2_-NT-CM-P[PPA] was fabricated, based on the fingerprint of the PPA polymer.

**Figure 6:**
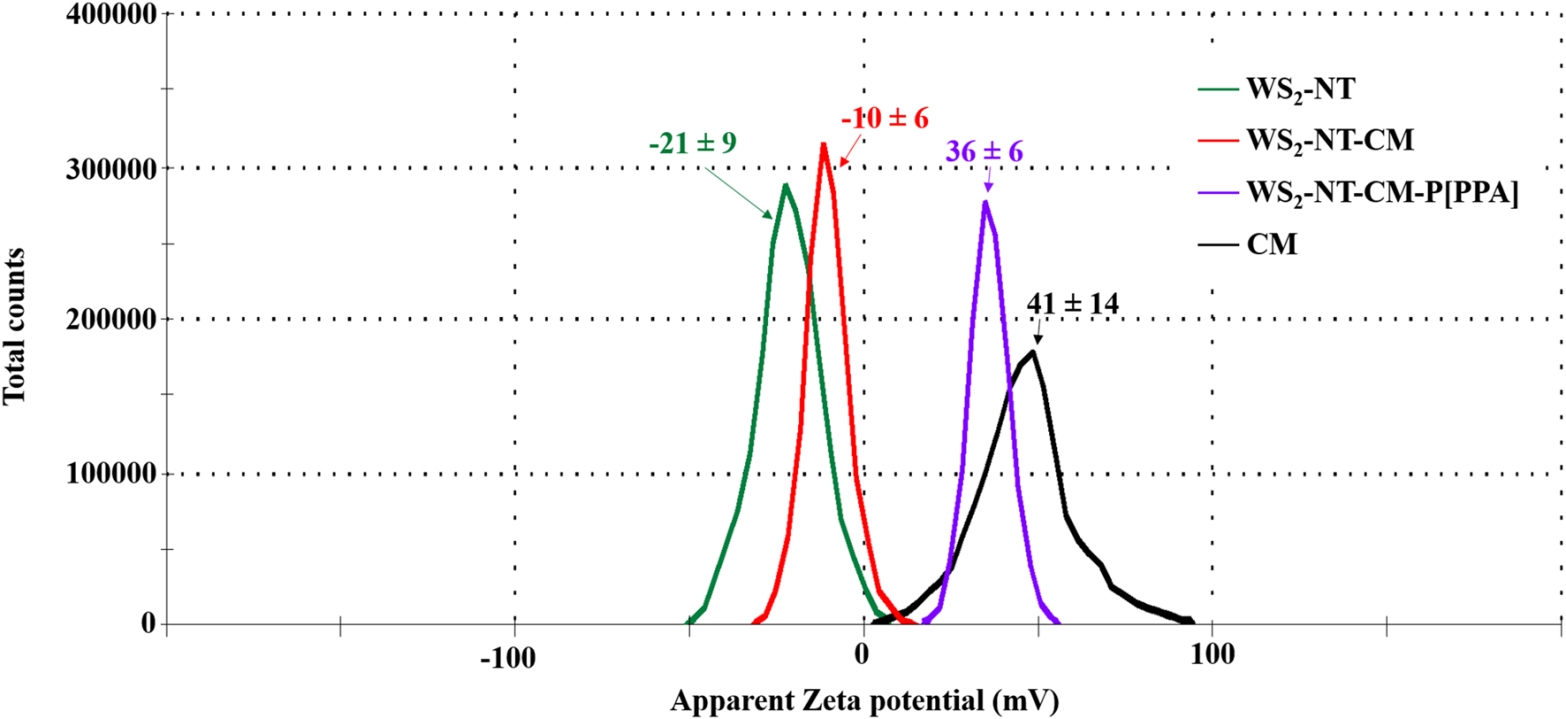
Zeta potential values and distribution curves of WS_2_-NT (green), WS_2_-NT-CM (red), WS_2_-NT-CM-P[PPA] (purple), and CM NPs (black). For each type of composite/particle, three measurements were performed, and values present the average result and the standard deviation.

**Figure 7:**
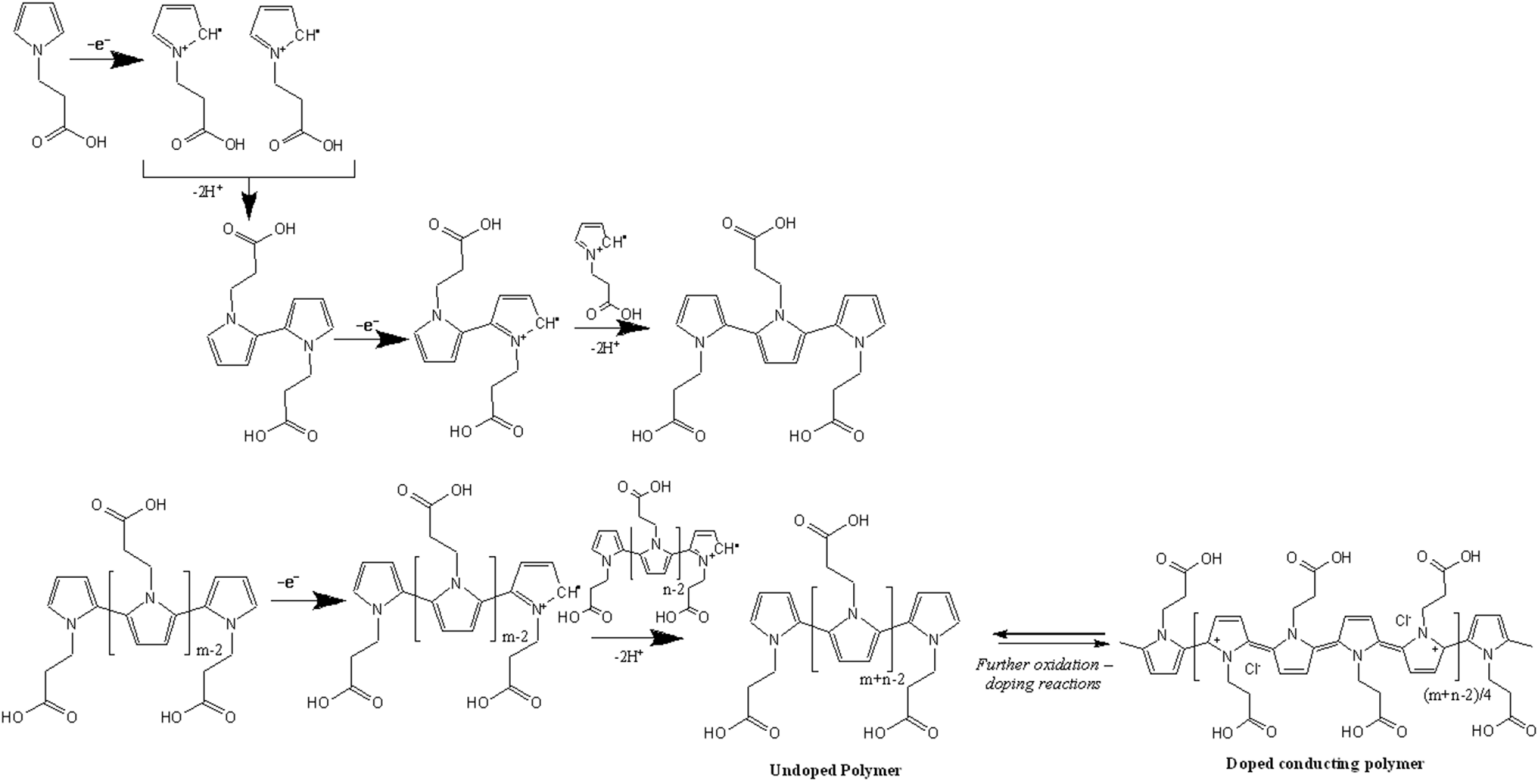
Proposed polymerization mechanism that explains the positive zeta potential of WS_2_-NT-CM-P[PPA] (this figure was drawn in ChemBioDraw Ultra 14.0 by Adept Scientific).

Figure 6 shows zeta potential averages and distribution curves for WS_2_-NTs, CAN-mag (CM), and their composites. Despite the scale-up in the synthesis of CM NPs and WS_2_-NT-CM, the measured zeta values (+ 41 ± 14 mV for CM and − 10.5 ± 6.4 mV for WS_2_-NT-CM) are very similar to the previous values: about + 41 mV for CM and − 9.9 mV for WS_2_-NT-CM. This demonstrates that the new large-scale synthesis protocols produce particles, which are comparable to those obtained by the previous protocols. Interestingly, the zeta potential of the new WS_2_-NT-CM-P[PPA] composite showed a high positive value (+ 36 mV) compared to WS_2_-NT-CM, despite the negative carboxylate groups of PPA. Apparently, these carboxylic acid moieties face the nanotubes and are involved in the coordination to the Ce, and thus have no influence on the surface zeta potential. Moreover, it is known that the zeta potential of polypyrroles is pH dependent; indeed, all zeta potential measurements of the four samples were performed in the pH range of 5–6. Within this range, the positive zeta potentials increase only slightly, as demonstrated by Zhang et al.^42^ however significant changes appear at pH below 3 and above 8. Therefore, in this work, the positive zeta potential value corresponds to the polymer backbone chain. During oxidative polymerization (chemical or electrochemical) of conjugated polymers such as polypyrroles, electrons are abstracted from the backbone of the polymer chain, creating p-type (positive) charge carriers (see Fig. 7). To maintain charge neutrality, some of the counter anions present in solution (i.e., Cl^−^ from the oxidant, FeCl_3_·6H_2_O) are incorporated into the growing polymer during polymerization. However, in aqueous media, the doped counter anions (Cl^−^) dissociate from the surface of the polymer and transfer into the bulk solution, leaving a positively charged surface and thereby a positive zeta potential.

**Figure 8:**
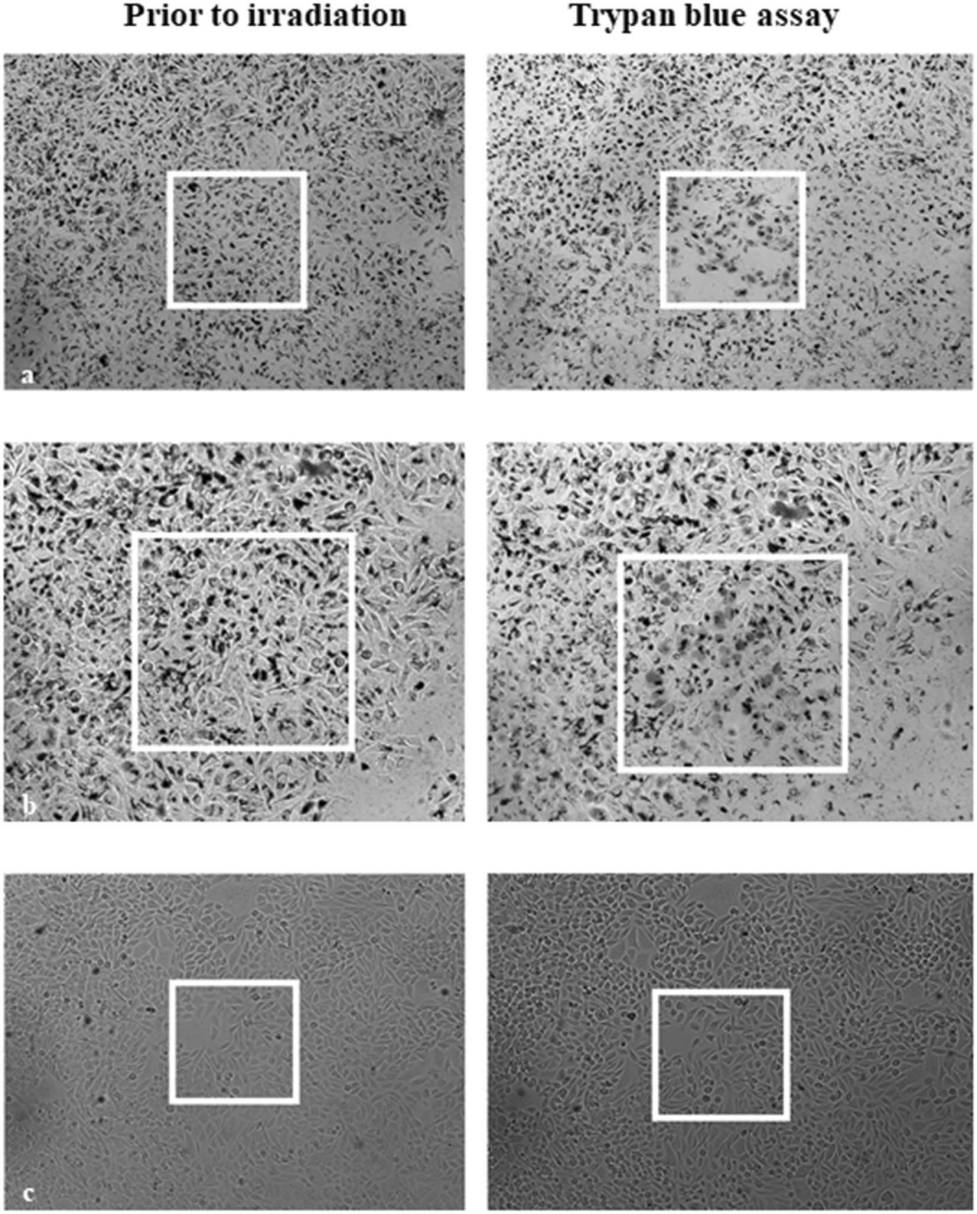
Phase-contrast microscopy images of HeLa cells. The white squares indicate 230 µm × 230 µm areas irradiated with a 808 nm laser. *Left column*: cells prior to near IR irradiation; *right column*: after irradiation for 31 s and application of trypan blue assay. Cells were pre-incubated with (**a**) WS_2_-NT-CM-P[PPA] or (**b**) WS_2_-NT-CM, (**c**) control.

In order to examine the potential of WS_2_-NT-CM and WS_2_-CM-P[PPA] as photothermal agents, human HeLa cells were incubated with both nanocomposites and treated with irradiation at 808 nm (IR laser) for 31 s. Figure 8 shows bright field microscopy images taken from a viability test of HeLa cells incubated for 14 h with WS_2_-NT-CM-P[PPA] (a), with WS_2_-NT-CM (b), and without any addition (c) as a control before (left) and after (right) irradiation. The irradiated area in each image is represented by a white square. It can be seen that after irradiation, the cells treated with either WS_2_-CM-P[PPT] or WS2-NT-CM appear gray and blurry after irradiation due to the collapse of the cell membrane and the penetration of the trypan blue dye. This phenomenon appears only within the irradiation limits (inside the square), but not for the untreated cells. This suggests that the cell death was not caused by irradiation alone or by the addition of the nanomaterials alone, but by the combination of both, proving photothermal activity. Some of the cells tested with WS_2_-NTs were detached during the viability test. Those cells were most likely dead as well, as detachment did not occur in the nontreated cells.

**Figure 9:**
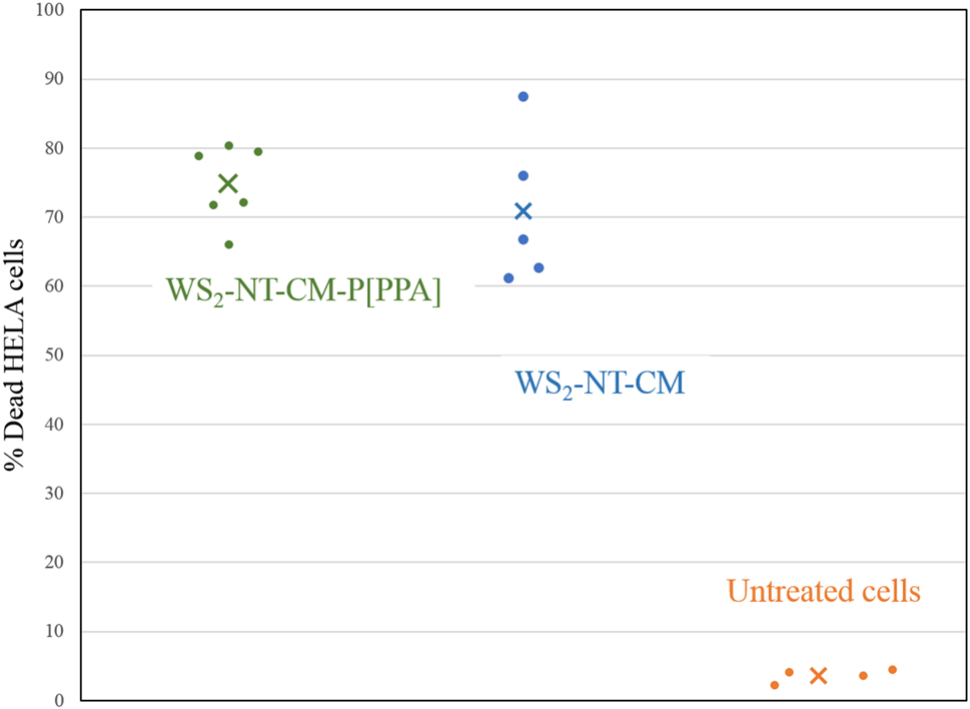
PTT results scatter plot for HeLa cancer cells incubated with WS_2_-NT-CM-P[PPA]—green series, WS2-NT-CM—blue series, and untreated cells for reference—orange series. Each dot represents one irradiated frame, the average number of dead cells after the irradiation is marked with an ‘ × ’. The cells were irradiated with an 808 nm near IR laser for 31 s at 90 mW.

In order to quantify the amount of dead cells after irradiation and to compare the two composites, live and dead cells were counted (Fig. 9). The average percentage of dead cells was 70.8% in WS_2_-NT-CM, 74.7% in WS_2_ -NT-CM-P[PPA], and 3.6% in the control (untreated) cells. From these experiments it is clear that the two composites give good results at 808 nm, with no statistically significant difference in killing efficiency. In comparison, black phosphorus reached killing efficiency of 40–60% at 285 nm and almost 100% at 380 nm^43^.

## Conclusion

A new core shell composite of WS_2_-pyrrole-N-propionic acid (PPA) was presented and evaluated for photothermal therapy (PTT). In addition, this work displays an improved protocol for the synthesis of both CM NPs and WS_2_-NT-CM composites in far greater amounts than in the recent publication^18^. The use of Ce-doped maghemite (CM) NPs solved the two major problems regarding functional polymerization on WS_2_-NTs: (1) the difficulty of polymerization onto an insoluble particle in most known solvents; and (2) the need for a linker between the monomers and the nanotubes preceding the polymerization. The CM NPs produce a much more stable nanotube suspension with strongly coordinating Ce sites on the NT surface, available for attachment of the polymerized monomers. The successful polymerization process is confirmed by TEM images and by TGA, FTIR, UV–visible spectroscopy, and zeta potential results, indicating a stable nanocomposite with high positive zeta potential value and a *core–shell* structure with 10–50 nm of polymer coating, which is 6.2% of the total composite (based on its weight loss in TGA).

The photothermal characterization of WS_2_ nanotubes was investigated for the first time, and their efficiency was calculated to be about 33%, in excellent agreement with WS_2_ nanosheets. In the PTT in vitro assay, we expected a larger effect with WS_2_-NT-CM-P[PPA], namely greater cell death after irradiation compared with uncoated WS_2_-NT-CM. Nonetheless, the addition of a polymer did not reduce the PTT activity. Both composites had a rather significant PTT effect, with about 70–75% cell death after only 31 s. Furthermore, the polycarboxylated polymer enables the linkage of numerous materials in a covalent chemical bond (especially drugs and biomolecules), thus making the polymerization process worthwhile. Additional materials may be added through the covalent chemical bond, including substances that target cancerous growth and other types of light-activated therapies such as photodynamic therapy. Owing to the magnetic CM middle phase, two additional benefits—high MRI imaging ability and magnetic delivery—are anticipated. Thus, these hybrid NTs have a high potential to act as a multidrug platform for targeted treatment while enabling imaging of the treated area. WS_2_ nanoplates and nanosheets shall be investigated.

## Data Availability

All data and materials support the published claims and comply with field standards. All research data are attached and are in concordance with disciplinary norms and expectations of the journal.
